# Evaluation of the Health Status of Indonesian Watersheds Using Impervious Surface Area as an Indicator

**DOI:** 10.3390/s23104975

**Published:** 2023-05-22

**Authors:** Rossi Hamzah, Bunkei Matsushita

**Affiliations:** 1 Graduate School of Life and Environmental Sciences, University of Tsukuba, Tennoudai 1-1-1, Tsukuba 305-8572, Japan; s1730209@u.tsukuba.ac.jp; 2 Research Center for Remote Sensing, National Research and Innovation Agency (BRIN), Bogor 16911, West Java, Indonesia; 3 Faculty of Life and Environmental Sciences, University of Tsukuba, Tennoudai 1-1-1, Tsukuba 305-8572, Japan

**Keywords:** temporal mixture analysis, MODIS NDVI, radiance-calibrated DMSP-OLS, SNPP-VIIRS-DNB, nighttime light

## Abstract

Impervious surfaces affect the ecosystem function of watersheds. Therefore, the impervious surface area percentage (ISA%) in watersheds has been regarded as an important indicator for assessing the health status of watersheds. However, accurate and frequent estimation of ISA% from satellite data remains a challenge, especially at large scales (national, regional, or global). In this study, we first developed a method to estimate ISA% by combining daytime and nighttime satellite data. We then used the developed method to generate an annual ISA% distribution map from 2003 to 2021 for Indonesia. Third, we used these ISA% distribution maps to assess the health status of Indonesian watersheds according to Schueler’s criteria. Accuracy assessment results show that the developed method performed well from low ISA% (rural) to high ISA% (urban) values, with a root mean square difference value of 0.52 km^2^, a mean absolute percentage difference value of 16.2%, and a bias of −0.08 km^2^. In addition, since the developed method uses only satellite data as input, it can be easily implemented in other regions with some modifications according to differences in light use efficiency and economic development in each region. We also found that 88% of Indonesian watersheds remain without impact in 2021, indicating that the health status of Indonesian watersheds is not a serious problem. Nevertheless, Indonesia’s total ISA increased significantly from 3687.4 km^2^ in 2003 to 10,505.5 km^2^ in 2021, and most of the increased ISA was in rural areas. These results indicate that negative trends in health status in Indonesian watersheds may emerge in the future without proper watershed management.

## 1. Introduction

The world population reached 8 billion in 2022 (UN 2022) [1]. A growing population requires more infrastructure (housing, office buildings, schools, hospitals, roads, parking lots, etc.) for human activity. As a result, the earth is gradually being paved with impervious surfaces [2,3,4]. Because impervious surfaces have different properties than natural land surfaces, their presence can alter the hydrological, ecological, and thermal characteristics of watersheds [5,6,7]. For example, Sohn et al.’s results show that impervious surfaces play an important role in reducing the likelihood of flooding, using the Texas watersheds as an example [6]. Kim et al. investigated the relationship between the impervious surface area percentage (ISA%) and water quality parameters in the Han River Basin in South Korea and concluded that ISA% is a suitable indicator to assess water quality in the basin [7].

In the 1990s, researchers began using ISA% values in watersheds to understand and evaluate the impact of impervious surfaces on watersheds. For example, Schueler suggested four categories representing watershed health levels according to ISA% values [8]. The four categories are: no impact (ISA% < 1%), stressed (I% ≤ ISA% < 10%), impacted (10% ≤ ISA% < 25%), and degraded (ISA% ≥ 25%). However, these criteria have so far been used to assess the health status of watersheds, especially at large (e.g., national, regional, and global) scales, mainly due to the difficulty in obtaining ISA% distribution maps of watersheds.

Satellite imagery has been widely used to estimate ISA% since the early 2000s [9,10,11,12,13,14,15,16,17,18,19]. This is due to its relatively low cost and suitability for large-area mapping. In this field, the Spectral Mixture Analysis (SMA) method is widely used for remote sensing of ISA% from satellite data with moderate spatial resolution [5,9,14,15,16,17]. However, the spectral complexity within the endmembers makes it difficult to accurately estimate ISA% from coarse spatial resolution images over large areas [10]. To address the above challenges, Yang et al. [10] proposed a Temporal Mixture Analysis (TMA) method for estimating ISA% from Moderate Resolution Imaging Spectroradiometer (MODIS) data. Compared to the widely used SMA-based method, the TMA-based method has two advantages: (1) it can significantly reduce the effects of endmember variability, and (2) it is suitable for large-scale ISA% mapping [10]. However, the original TMA-based method often misclassified bare land as impervious surfaces and thus overestimated ISA% [12]. To solve this overestimation problem, Pok et al. [12] developed a new method by combining daytime satellite data (MODIS) with nighttime satellite data (Defense Meteorological Satellite Program-Operational Linescan System: DMSP-OLS). In Pok’s method, the original DMSP-OLS nighttime light (NTL) data were converted to the Enhanced vegetation index Adjusted Nighttime Light Index (EANTLI) to mitigate blooming and saturation effects in the original stable light data [20]. However, due to the very sensitive relationship (natural logarithmic function) between ISA% and EANTLI in rural areas (ISA% < 40%), the use of EANTLI is prone to large errors in estimating ISA% in these areas. These errors cannot be ignored when estimating the total ISA% for a watershed [21]. This is because watersheds typically have a much higher number of pixels with low ISA% values (rural areas) than pixels with high ISA% values (urban areas). Therefore, it can be argued that the overly sensitive ISA%-EANTLI relationship in rural areas obtained by Pok et al. [12] will likely lead to misassessments of watershed health status when using Schueler’s criteria.

Meanwhile, the Suomi National Polar-orbiting Partnership (SNPP) satellite was launched in late 2011. The Visible Infrared Imaging Radiometer Suite (VIIRS) sensor with specific panchromatic Day and Night Band (DNB) onboard the SNPP has collected global daily NTL data [22] and has provided an annual time series of global SNPP-VIIRS-DNB-NTL data since 2012 [23]. More importantly, the SNPP-VIIRS-DNB-NTL product does not saturate in urban areas and has a less blooming effect because they are radiometrically calibrated and sensitive to lower light levels [24]. For the period prior to 2012, another global NTL product was generated for seven years (1996, 1999, 2000, 2003, 2004, 2006, and 2010) based on the pre-flight DMSP-OLS calibration [25]. The radiance-calibrated DMSP-OLS-NTL product is also free of saturation and blooming issues. Therefore, these two NTL products offer new opportunities to solve the problem of Pok et al. [12] by rebuilding a more robust relationship between ISA% and NTL and improving the accuracy of ISA% estimation in rural areas.

Consequently, using Indonesia as an example, the objectives of this study are to: (1) develop a method for estimating ISA% on a national scale based on daytime (MODIS) and nighttime (radiance-calibrated DMSP-OLS-NTL and SNPP-VIIRS-DNB-NTL) satellite data; (2) generate an annual distribution map of ISA% for Indonesia from 2003 to 2021; and (3) assess the health status of all watersheds in Indonesia between 2003 and 2021 using Schueler’s criteria.

## 2. Materials and Methods

### 2.1. Study Area and Datasets

**Figure 1:**
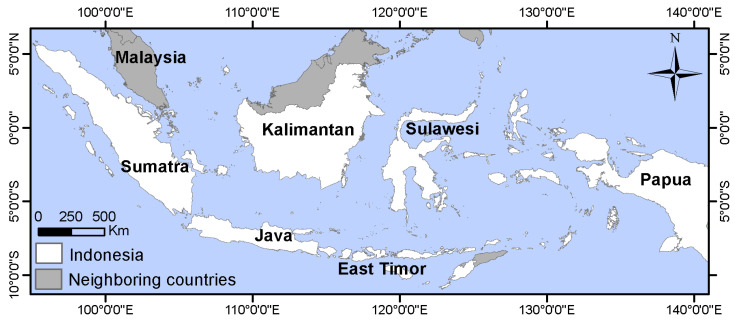
Location of the study area.

Indonesia (11° S–6° N, 95° E–141° E), with an area of 1,904,569 km^2^, is our study area (Figure 1). Indonesia has 17,500 islands scattered on either side of the equator, with more than 7000 uninhabited. Sumatra, Kalimantan, and Papua occupy nearly three-quarters of Indonesia’s area. The rest of Indonesia mainly consists of East Timor, Maluku, Java, and Sulawesi [26]. Indonesia’s population grew from 87.75 million in 1960 to 273.75 million in 2021, making it the fourth most populous country in the world [27,28]. The population of Indonesia is mostly concentrated on Java Island. However, other islands include several heavily populated areas, such as Medan City (Sumatra), Makassar City (Sulawesi), and Banjarmasin City (Kalimantan) [29].

Indonesia is one of the fastest-growing economies and the largest in Southeast Asia. Indonesia’s GDP growth averaged 4.9% annually from 2000 to 2021, with 5.02%, −2.07%, and 3.69% in 2019, 2020, and 2021, respectively [30]. Moreover, Indonesia’s annual infrastructure budget increased eightfold from Rp 50 billion in 2003 to 400 billion in 2021 [31,32,33].

Indonesia’s climate, with dry and wet seasons, is almost entirely tropical, hot and humid, but milder in the highlands [34]. The average annual precipitation falls between 180 and 280 mm per month, with the largest precipitation occurring between October and January [35]. The dominant landscapes in Indonesia are dense forests (51.7%), agricultural land (31.2%), and others (17.1%) in 2018 [29].

Five datasets were used in this study (Table 1). The first dataset is the MODIS 16-day composited Normalized Difference Vegetation Index (NDVI, MOD13A2), which has 23 NDVI composites for each year from 2000 to 2021. This dataset can provide temporal information about the land surface with a spatial resolution of 1 km. However, 2000–2002, 2005, 2007–2009, and 2011 NDVI composites were not used due to the unavailability of the corresponding second dataset. All annual NDVI composites were smoothed using Savitzky–Golay filter-based method to further improve data quality (i.e., further reduced the effects of cloud/noise effects remaining on the NDVI time series) [36]. The smoothed NDVI data were then sorted in ascending order, and only the last 12 largest NDVI composites were used to estimate non-vegetation fraction according to previous studies [10,11,12]. In addition, all water pixels were pre-masked to avoid misclassification to non-vegetation and save computation time.

The second dataset is the global DMSP-OLS radiance-calibrated NTL time series with inter-calibration, which records the annual average radiance calibrated with a cell size of 1 km × 1 km for 1996, 1999, 2000, 2003, 2004, 2006, and 2010 [25]. However, the 1996, 1999, and 2000 data were not used due to the unavailability of the corresponding first dataset. A shift-based method was employed to eliminate the geometric errors in DMSP-OLS data [37], and inter-calibration was implemented using coefficients provided by NOAA [25]. This dataset is generally considered to be free of saturation and blooming effect problems in nighttime lights [25,38] and was used to replace the global DMSP-OLS stable light product that has been widely used in previous studies [12,13,17,39,40].

The third dataset is an annual time series of global SNPP-VIIRS-DNB-NTL data from 2012 to 2021 (version 2) [23,41,42]. All SNPP-VIIRS-DNB-NTL data were resampled from 750 m to 1km to match the second dataset. A Gaussian low-pass filter was then applied to all SNPP-VIIRS-DNB-NTL data to make the spatial details of the filtered SNPP-VIIRS-DNB-NTL images more similar to those of the DMSP-OLS data. A window size of 5 × 5 pixels and a standard deviation value of 1.75 were applied to the Gaussian filter [43]. This dataset is also used to replace the global DMSP-OLS stable light product in this study, as it also mitigates saturation and blooming issues in NTL data. Both radiance-calibrated DMSP-OLS-NTL and SNPP-VIIRS-DNB-NTL data are available from Earth Observation Group (EOG) from the Colorado School of Mines (https://eogdata.mines.edu accessed on 1 April 2021) [41,44,45]. The second and third datasets were used to build a relationship between ISA% and NTL by combining the results obtained from the first dataset to reduce the effects of bare land.

The fourth dataset is the watershed polygons of Indonesia, which were obtained from the Hydrological data and maps based on SHuttle Elevation Derivatives (HydroSHEDS, version 1) [46,47]. HydroSHEDS has 12 levels to draw watersheds with different sizes. This study selected level 7, with an average watershed area of 2463 km^2^ in Indonesia, following previous studies [48]. This dataset was used to estimate the ISA% for each watershed in Indonesia.

The fifth dataset is Google Earth imagery, collected in 2003–2004, 2006, 2010, and 2012–2018, with a spatial resolution of 0.5 m. This dataset was used to provide reference ISA% samples for accuracy assessment. A total of 150 samples were collected with a 3 km by 3 km sampling window, considering the ISA% values evenly distributed over the full dynamic range (i.e., ISA% values from 0 to 100%). A visual digitizing method was used to classify each reference sample as impervious surface or non-impervious surface in ArcMap software (version 10.8.1, licensed under the University of Tsukuba, Tsukuba, Japan).

### 2.2. Method

#### 2.2.1. Estimating Non-Vegetation Fraction from MODIS-NDVI Time Series

This study employed the TMA method to estimate the non-vegetation fraction from the MODIS-NDVI time series. In the TMA method, the NDVI temporal profile of a mixed pixel (${NDVI}_{mix}$) is considered to be the linear combination of NDVI temporal profiles of the identified endmembers, and can be mathematically written as [10]:

(1) $${NDVI}_{mix}=\sum_{i=1}^{n}f_{i}{NDVI}_{i}+\varepsilon ,$$

where ${NDVI}_{i}$ is the NDVI temporal profile of endmember i, $f_{i}$ is the fraction of endmember i, n is the number of endmembers, and ε is the residual representing the model error. The fractions of the endmembers are nonnegative, and their sum equals 1, as defined in Equation (2):

(2) $$\sum_{i=1}^{n}f_{i}=1,f_{i}\geq 0.$$

To solve Equation (1), we used the Fully Constrained Least Squares (FCLS) method coded in the Python programming language using Pysptools [49,50].

**Figure 2:**
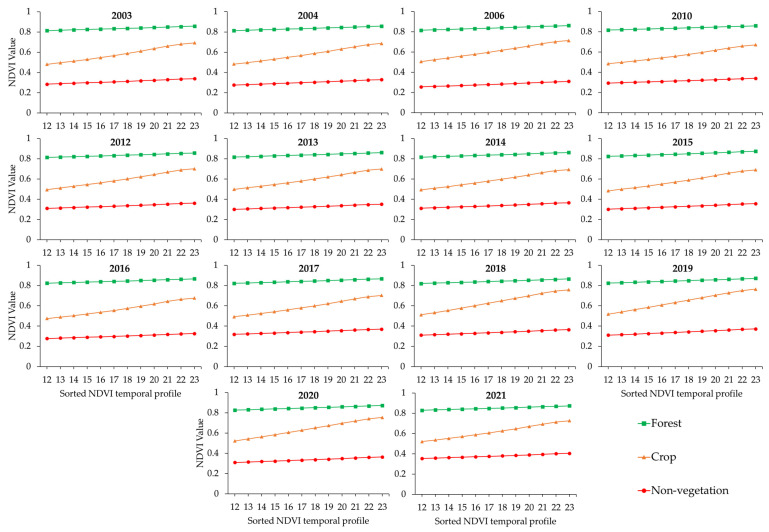
NDVI temporal profiles of selected endmembers for each year.

We identified three endmembers (forest, crop, and non-vegetation) by carrying out a minimum noise fraction (MNF) transform for the last 12 largest NDVI values [12]. Non-vegetation represents ISA, bare land, or a mixture thereof. According to Yang et al. [10], forest pixels should have low NDVI temporal profile gradients and high NDVI values; crop pixels should have high NDVI temporal profile gradients and high NDVI values; and non-vegetation pixels should have low NDVI temporal profile gradients and low NDVI values. All identified forest, crop, and non-vegetation pixels were averaged as corresponding endmembers. Figure 2 shows the NDVI temporal profiles of the selected endmembers for each year.

#### 2.2.2. Generation of Consistent NTL Time Series from 2003 to 2021

**Figure 3:**
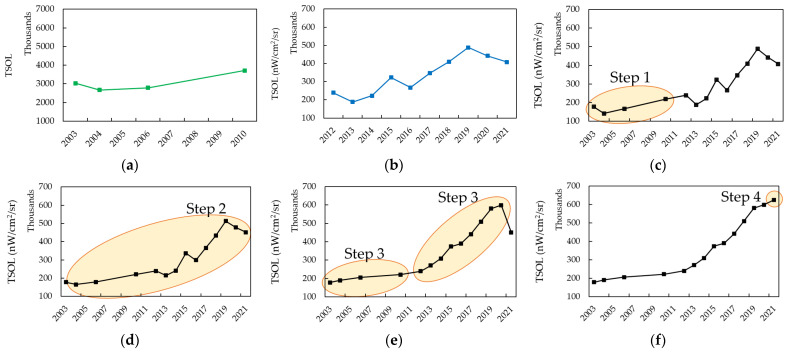
The total sum of light (TSOL) of NTL time series in the stepwise calibration: (**a**) original radiance-calibrated DMSP-OLS-NTL time series; (**b**) original SNPP-VIIRS-DNB-NTL time series; (**c**) step 1: VIIRS-like NTL time series (2003, 2004, 2006, and 2010) and original SNPP-VIIRS-DNB-NTL time series (2012–2021); (**d**) step 2: NTL time series after zero value correction; (**e**) step 3: NTL time series after inter-annual series correction; and (**f**) step 4: NTL time series after adjustment of 2021 SNPP-VIIRS-DNB-NTL data. The shaded ellipses are calibrated with NTL data in each step.

We generated consistent NTL time series from 2003 to 2021 by integrating the radiance-calibrated DMSP-OLS-NTL data (2003, 2004, 2006, and 2010) and SNPP-VIIRS-DNB- NTL data (2012–2021). For this, we adopted a stepwise calibration method [51]. This method involves four steps in sequence, and Figure 3 shows the correction results for each step.

*Step 1: Generating VIIRS-like NTL data from radiance-calibrated DMSP-OLS-NTL data for 2003, 2004, 2006, and 2010*. This was performed as the SNPP-VIIRS-DNB-NTL data provide better spatial, temporal, and radiometric resolutions than do the radiance-calibrated DMSP-OLS-NTL data. Here, we used the 2010 radiance-calibrated DMSP-OLS-NTL image and the 2012 SNPP-VIIRS-DNB-NTL to obtain inter-calibration coefficients, assuming no nighttime light changes between 2010 and 2012. We selected pixels with stable light based on their coefficient of variation (CV) within a moving window size of 3 × 3 pixels in the two NTL images. Pixels with CV values below 20% in both NTL images were considered NTL stable regions [52,53]. A cubic polynomial regression model was then constructed based on these extracted light stable pixel pairs (N = 31,168) to obtain the relationship between radiance-calibrated DMSP-OLS-NTL and SNPP-VIIRS-DNB-NTL data, as written in Equation (3):

(3) $${VIIRS}_{2012}=a_{0}{\left({DMSP}_{2010}\right)}^{3}+a_{1}{\left({DMSP}_{2010}\right)}^{2}+a_{2}({DMSP}_{2010})+a_{3};$$

where ${DMSP}_{2010}$ represents the 2010 radiance-calibrated DMSP-OLS-NTL; ${VIIRS}_{2012}$ represents the 2012 SNPP-VIIRS-DNB-NTL; and $a_{0}$, $a_{1}$, $a_{2}$, and $a_{3}$ are 1.98 × 10^−7^, −0.000241, 0.143725, and −0.616852, respectively (R^2^ = 0.90 and RMSD = 1.52 mW/cm^2^/sr). After the correction in step 1, the two NTL datasets were comparable and consistent (Figure 3c).

*Step 2: Zero value correction*. We then performed zero value correction on all NTL time series to further improve their compatibility and continuity, assuming that lit pixels do not disappear in the NTL images. In other words, the lit pixels of this year should not decrease to zero next year. The equation is shown as follows [54]:

(4) $${DN}_{\left(n,i\right)}=\left\{\begin{array}{cc} {DN}_{\left(n-1,i\right)} & \left({DN}_{\left(n,i\right)}=0)\;\&\;{(DN}_{\left(n-1,i\right)}>0\right)\\ {DN}_{\left(n,i\right)} & otherwise \end{array}\right.;$$

where ${DN}_{\left(n-1,i\right)}$ and ${DN}_{\left(n,i\right)}$ are the NTL values of the *i*th lit pixel in the *n*−1th and *n*th years, respectively. Figure 3d shows the correction results at this step.

*Step 3: Inter-annual series correction*. The discontinuity effect was still present between annual NTL images after correction using Equation (4). Therefore, an inter-annual series correction was performed using Equation (5) to eliminate abnormal fluctuations [54,55,56,57,58]. This processing assumes that there is no dimming of lit pixels in the NTL time series. Note that the inter-annual series correction was performed separately for the DMSP-OLS (2003, 2004, 2006, 2010) and VIIRS-DNB (2012–2021) NTL data. This is to preserve the original quality of the SNPP-VIIRS-DNB-NTL data from the influence of the radiance-calibrated DMSP-OLS-NTL data. The Equation (5) is written as [54]:

(5) $${DN}_{\left(n,i\right)}=\left\{\begin{array}{cc} 0 & {DN}_{\left(n+1,i\right)}=0\\ {DN}_{\left(n-1,i\right)} & {(DN}_{\left(n+1,i\right)}>0)\;\&\;{(DN}_{\left(n-1,i\right)}>{DN}_{\left(n,i\right)})\\ {DN}_{\left(n,i\right)} & otherwise \end{array}\right.;$$

where ${DN}_{\left(n-1,i\right)}$, ${DN}_{\left(n,i\right)}$, and ${DN}_{\left(n+1,i\right)}$ are the NTL values of the *i*th lit pixel in the *n*−1th, *n*th, and *n*+1th years, respectively. Figure 3e shows the correction results at this step.

*Step 4: Adjustment of 2021 SNPP-VIIRS-DNB-NTL data.* Since Equation (5) cannot correct the NTL time series for the last year, we used Equation (6) [37]:

(6) $${DN}_{\left(k,i\right)}=\left\{\begin{array}{cc} {DN}_{\left(k-1,i\right)} & {DN}_{\left(k-1,i\right)}>{DN}_{\left(k,i\right)}\\ {DN}_{\left(k,i\right)} & otherwise \end{array}\right.;$$

where ${DN}_{\left(k-1,i\right)}$ and ${DN}_{\left(k,i\right)}$ are the NTL values of the *i*th lit pixel for 2020 and 2021, respectively. Figure 3f shows the final correction results for the NTL time series.

In general, countries that have not experienced serious situations such as political instability, economic collapse, or natural disasters do not show a downward trend in the light of that country [58,59,60]. Nevertheless, all assumptions for processing NTL time series may not be suitable for real NTL changes (e.g., COVID-19 decreased NTL globally), but are useful for ISA% estimation, as the disappearance of an existing impervious surface is considered to be rare. The corrected NTL time series is denoted as NTL_corrected_ hereafter.

#### 2.2.3. Building Relationships between ISA% and NTL_corrected_

**Figure 4:**
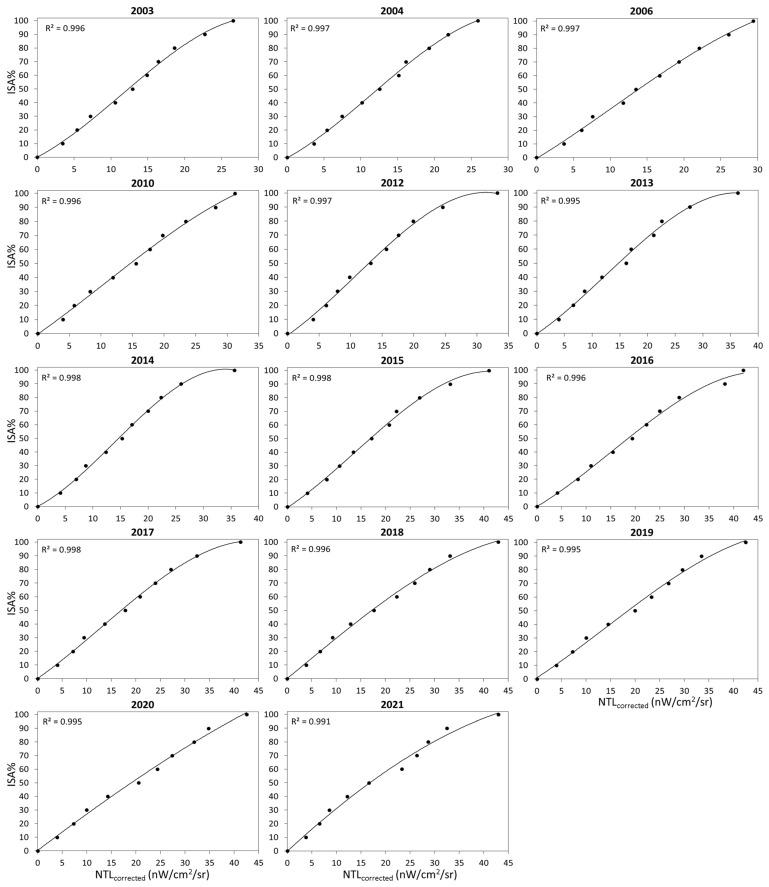
Relationships between NTL_corrected_ and ISA% for each year.

In this study, we followed the method proposed by Pok et al. [12] to build the relationship between NTL_corrected_ and ISA% for each year. First, the non-vegetation fraction map of each year (taken from Section 2.2.1) was evenly divided into 10 groups (i.e., 1–10%, 11–20%, 21–30%, 31–40%, 41–50%, 51–60%, 61–70%, 71–80%, 81–90% and 91–100%). Second, the NTL_corrected_ values were statistically analyzed to find the upper limit of the 95th percentile for each non-vegetation fraction group (see Figure S1 for reference). The NTL_corrected_ value at the 95th percentile line is considered to correspond to ISA% for this group. Therefore, we used all NTL_corrected_ values at the 95th percentile line and the corresponding ISA% values to build the relationship between ISA% and NTL_corrected_ for each year. The results are shown in Figure 4 and Table 2. All relationships were found to have coefficient of determination values greater than 0.99 and RMSD values less than 3%.

#### 2.2.4. Assessing the Health Status of Indonesian Watersheds

Using the relationships established in Section 2.2.3, we generated preliminary distribution maps of ISA% in Indonesia for each year from the corresponding NTL_corrected_ maps generated in Section 2.2.2. These preliminary ISA% maps were then compared to corresponding non-vegetation maps generated in Section 2.2.1, and smaller values were selected to generate final distribution maps of ISA% for each year. The reasons for this were that non-vegetation land cover includes both ISA and bare land, and ISA% values must be less than or equal to the values of non-vegetation fraction [12,21]. Finally, we assessed the health status of Indonesian watersheds annually based on the criteria proposed by Schueler [8]. The classification criteria are: the “no impact” category with an ISA% value of the watershed less than 1%, the “stressed” category with an ISA% value of the watershed between 1% and 10%, the “impacted” category with an ISA% value of the watershed between 10% and 25%, and the “degraded” category with an ISA% value of the watershed greater than 25% [8,61,62].

#### 2.2.5. Accuracy Assessment

Three indices were used to evaluate the developed method’s performance: the root mean square difference (RMSD), the mean absolute percentage difference (MAPD), and the bias. These indices are defined as follows:

(7) $$RMSD=\sqrt{\frac{\sum_{i=1}^{N}{\left({ISA}_{est,i}-{ISA}_{ref,i}\right)}^{2}}{N}},$$

(8) $$MAPD=\frac{1}{N}\sum_{i=1}^{N}\left|\frac{{ISA}_{est,1}-{ISA}_{ref,i}}{{ISA}_{ref,i}}\right|\times 100\%,$$

and

(9) $$Bias=\frac{\sum_{i=1}^{N}{ISA}_{est,1}-{ISA}_{ref,i}}{N},$$

where ${ISA}_{est,i}$ refers to the ith estimated ISA% value, ${ISA}_{ref,i}$ represents the *i*th reference ISA% value, and *N* is the total number of samples. Furthermore, the proposed method was compared with the global artificial impervious area (GAIA) dataset [63]. The coefficient of determination (R^2^) between ${ISA}_{est,i}$ and ${ISA}_{ref,i}$ was also calculated.

## 3. Results

### 3.1. Annual ISA% Distribution Maps in Indonesia

**Figure 5:**
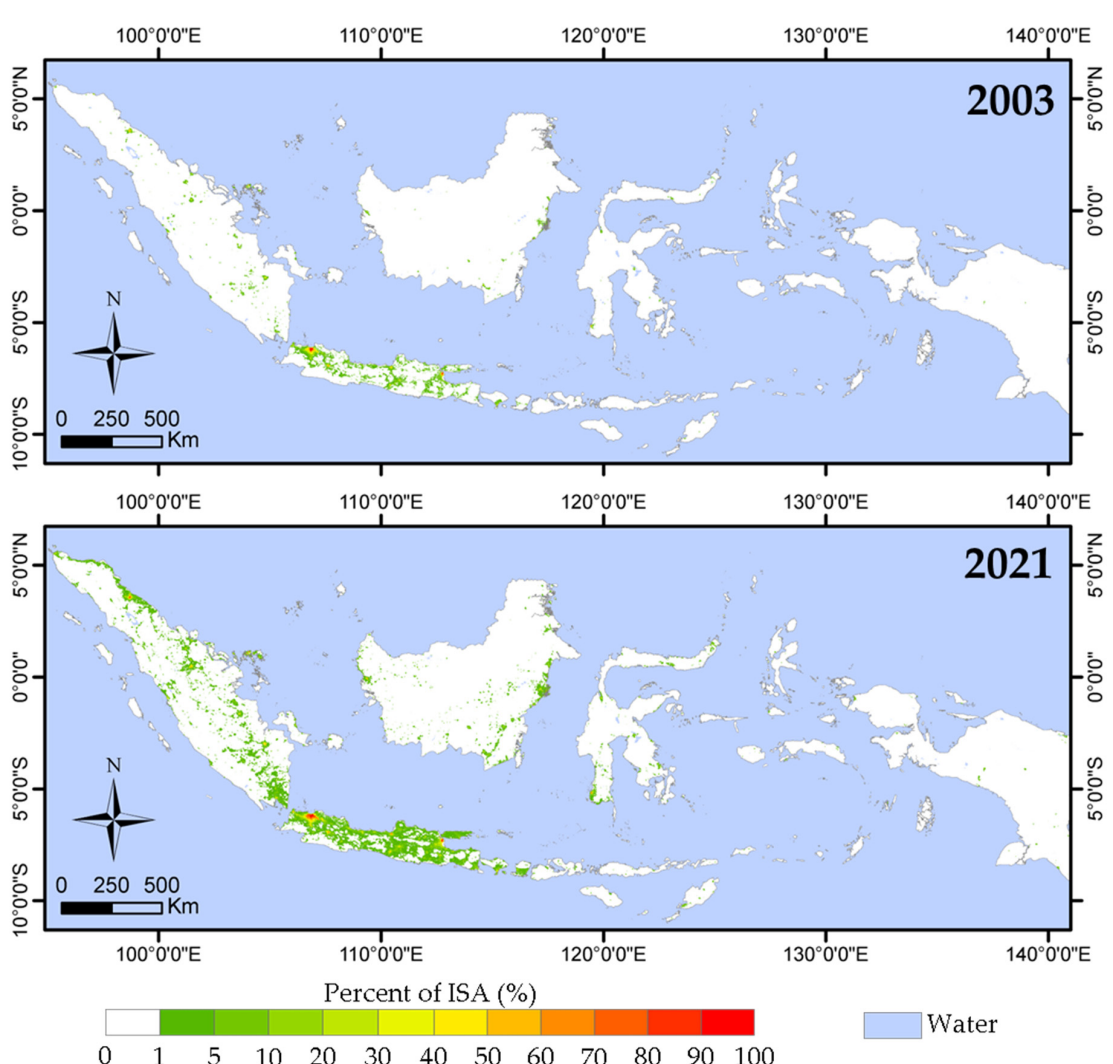
Distribution maps of ISA% in Indonesia. (**top**) 2003, (**bottom**) 2021. See Figure S2 for all years.

Figure 5 shows the annual distribution map of ISA% for 2003 and 2021 generated using the method described in Section 2.2. The total ISA for each year obtained from the annual ISA% distribution map is shown in Table 3. Spatially, the high ISA% values are mainly distributed in Java Island and Sumatra Island, especially in two large cities of Java Island (Jakarta in the east and Surabaya in the west). In contrast, Kalimantan Island, Sulawesi Island, and western Papua have relatively low ISA% values.

In terms of change over time, the total ISA in Indonesia showed a significant increasing trend over the study period (R^2^ = 0.84, *p* < 0.001). There was an average increasing rate of 378.79 km^2^/year, as the total ISA was 3687.35 km^2^ (0.2% of Indonesia’s land surface) in 2003 and increased to 10,505.5 km^2^ (0.6% of Indonesia’s land surface) in 2021. A turning point in the rise of the ISA values was observed in 2016. Between 2003 and 2016, the total ISA in Indonesia increased gradually from 3687.35 km^2^ to 4931.67 km^2^ with an average increasing rate of 95.72 km^2^/year. In contrast, between 2016 and 2021, the total ISA in Indonesia increased rapidly from 4931.67 km^2^ to 10,505.5 km^2^, with an average increasing rate of 1114.77 km^2^/year (nearly a 12-fold increase).

### 3.2. Performance of the Developed Method

**Figure 6:**
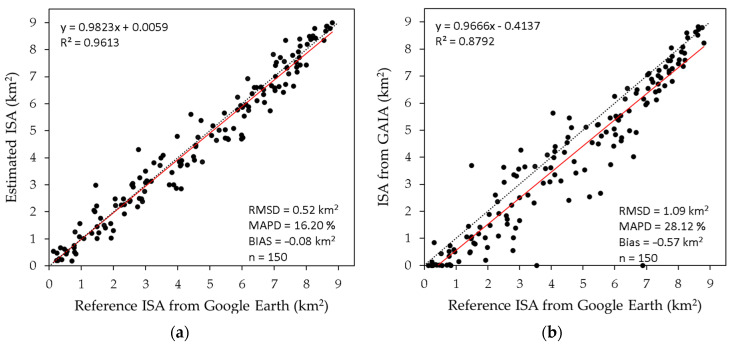
Accuracy assessment results. (**a**) Estimated ISA by the proposed method; (**b**) Estimated ISA from the GAIA dataset [63]. Reference ISA values were obtained by visual interpretation of the corresponding Google Earth imagery. The dashed black and the solid red lines represent the 1:1 line and regression line, respectively.

Figure 6a shows the accuracy assessment results of the proposed method. The MAPD and RMSD of the proposed method were 16.2% and 0.52 km^2^, respectively. A slight underestimation was also observed (bias = −0.08 km^2^). With an R^2^ value of 0.96, the proposed method explained 96% of the variance in the ISA estimations from Google Earth images.

The accuracy assessment for the GAIA dataset is also shown for comparison (Figure 6b). Compared to the accuracy of the proposed method, the ISA estimations from the GAIA dataset show a large difference from the values of Google Earth images, with an RMSD of 1.09 km^2^ and a MAPD of 28.12%, as well as a large underestimation (Bias = −0.57 km^2^). Notably, some impervious surfaces were not detected in the GAIA dataset but were adequately estimated by the proposed method (see points on the horizontal axis in Figure 6b).

### 3.3. Watersheds Evaluation in Indonesia

**Figure 7:**
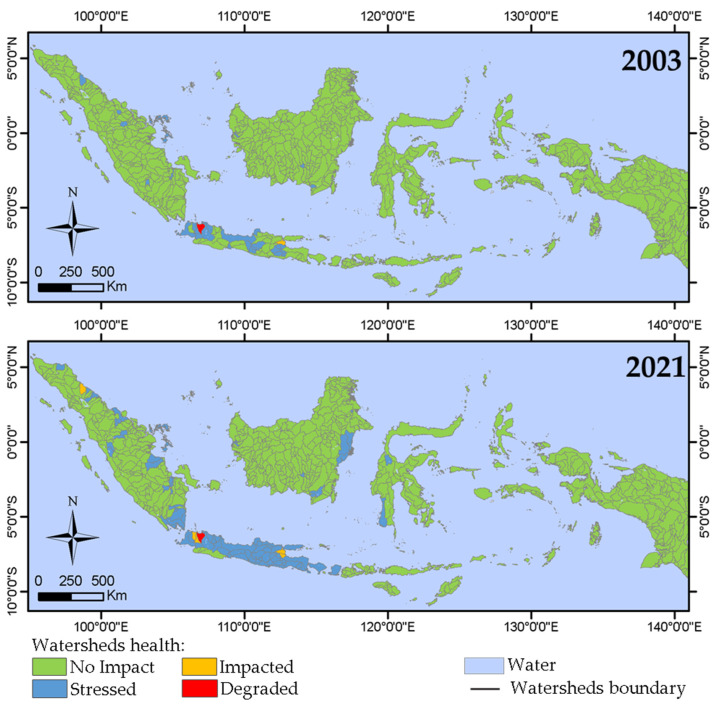
Distribution maps of watershed health status in Indonesia. (**top**) 2003, (**bottom**) 2021. See Figure S3 for all years.

**Figure 8:**
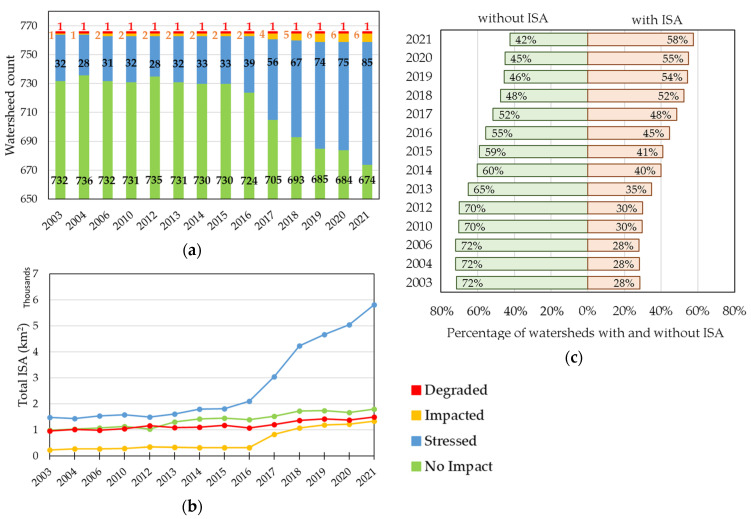
Statistical results for each year (2003–2021). (**a**) Number of watersheds per category; (**b**) Total ISA per category; (**c**) Percentage of watersheds with and without ISA.

The total ISA% (ratio of total ISA to the watershed area) for each watershed was calculated by combining the annual ISA% distribution map produced in Section 3.1 with the watershed polygon data obtained from HydroSHEDS. These calculated ISA% values were then used to classify all watersheds into four categories (i.e., no impact, stressed, impacted, and degraded) based on Schueler’s criteria [8]. Figure 7 shows the classification map for 2003 and 2021, and Figure 8 shows the statistical results for each year.

In 2003, 95.6% (732 out of 766) of Indonesian watersheds were in the no impact category (Figure 8a), and 72% had no ISA detected (Figure 8c). Among the remaining 4.4% of Indonesian watersheds, 32 (4.2%), 1 (0.1%), and 1 (0.1%) belonged to the stressed, impacted, and degraded categories, respectively. Furthermore, in 2003, most of the degraded, impacted, and stressed watersheds were distributed in Java Island (Figure 7). However, the number of no impact watersheds decreased to 674 (88% of all watersheds in Indonesia) by 2021. Conversely, by 2021, the number of stressed and impacted watersheds increased to 85 (11.1%) and 6 (0.8%), respectively. Although the number of degraded watersheds remained unchanged from 2003 to 2021, the area of the ISA increased by 537.4 km^2^ during this period (Figure 8b). Moreover, the number of no impact category decreased (from 732 in 2003 to 674 in 2021), but its area almost doubled from 989.8 km^2^ to 1802.6 km^2^ (Figure 8b). This is because the percentage of watersheds without ISA reduced from 72% to 42% (Figure 8c).

Moreover, in 2021, almost all watersheds in Java Island fell into the stressed category, with Sumatra, Kalimantan, and Sulawesi showing a clear increase in the stressed category (Figure 7b).

## 4. Discussion

### 4.1. Improved Relationships between ISA% and NTL Data

In this study, we developed a method to estimate ISA% based on daytime and nighttime satellite data. The biggest difference compared to previous studies [12,21] is the use of the radiance-calibrated DMSP-OLS-NTL and SNPP-VIIRS-DNB-NTL data instead of EANTLI data to generate the NTL time series. This improvement allows us to obtain a more reasonable relationship between ISA% and NTL data for each year (Figure 4). Pok et al.’s study found two relationships between ISA% and EANTLI: (1) the natural logarithmic function relationship in rural areas, and (2) the quadratic order polynomial function relationship in urban areas. Two functions required in their study are likely caused by the use of EANTLI. The formula designed to calculate EANTLI from the normalized DMSP-OLS-NTL and the Enhanced Vegetation Index (EVI) compresses the NTL dynamic range in rural areas, but expands it in urban areas [20]. Moreover, using the natural logarithmic function relationship in rural areas makes the estimation of ISA% in these areas very sensitive to variations in NTL, which tends to lead to large errors in the estimation of ISA%. The above two issues were resolved in this study by using high-quality NTL data (i.e., radiance-calibrated DMSP-OLS-NTL and SNPP-VIIRS-DNB-NTL data). From Figure 4, we can see that only one cubic polynomial function is needed to represent the relationship between ISA% and NTL across the study area for each year. These results indicate the importance of using high-quality NTL data.

We also found that the ISA%-NTL relationship varied between research years (Figure 4 and Table 2). This is likely due to the fact that the quality of MODIS-NDVI and NTL data can vary from year to year due to weather conditions and sensor degradations in that year. In addition, the assumptions and corrections for generating the NTL time series can also lead to some unrealistic situations during the year. Therefore, building the ISA%-NTL relationship year-to-year helps to generate a more accurate annual ISA% distribution map.

All inputs required for the developed method can be obtained from each of the published global products. This facilitates the implementation of the developed method in other regions, by rebuilding a more appropriate ISA%-NTL relationship. Unfortunately, the SNPP-VIIRS-DNB-NTL data are only available after 2012, and the radiance-calibrated DMSP-OLS-NTL can only go back to 1996 for seven years. Due to this limitation, it remains difficult to generate long-term ISA% distribution maps (e.g., over 30 years).

Another potential limitation of the proposed method is that the ISA% within a pixel will be underestimated if it is dark or light is absent, even though the pixel is covered with an impervious surface. However, this seems to be a rare case, compared to lit impervious surface pixels.

### 4.2. Reliability of the Generated ISA% Distribution Maps

**Figure 9:**
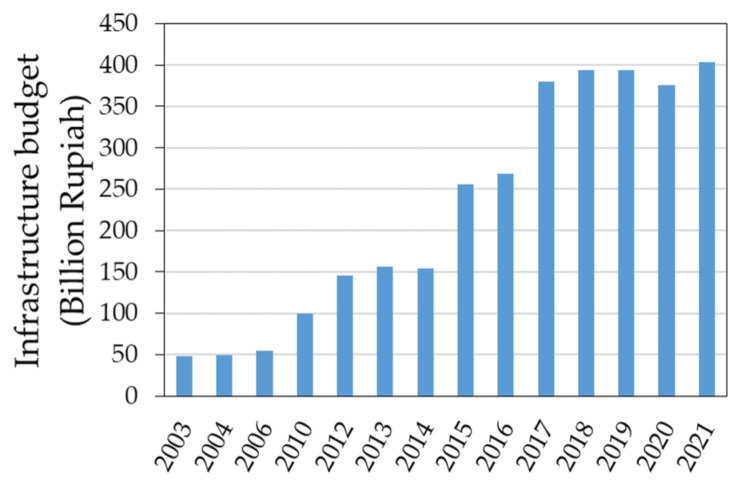
Indonesia’s infrastructure budget from 2003 to 2021.

Gong et al. (2020) published the GAIA dataset generated from Landsat imagery acquired between 1985 and 2018. This dataset contains 34 annual distribution maps with a spatial resolution of 30 m, and each pixel provides information on whether it is an impervious surface (yes or no). We also calculated the total ISA for Indonesia from 2003 to 2018 using the GAIA dataset and compared it to the results obtained in this study (Table 3). From Table 3, we can see that the total annual ISAs estimated from the GAIA dataset are all higher than those from the ISA% maps generated in this study, except for 2018. Additionally, we did not find that the total ISA areas increased significantly from 2016 to 2017 from the GAIA-based results. This is different compared to the results found in the ISA% distribution maps generated in this study. To see which finding makes more sense, we investigated Indonesia’s infrastructure budget from 2003 to 2021 (Figure 9) [31,32,33]. Figure 9 also shows that Indonesia’s infrastructure budget increased significantly from 2003 to 2021 (especially from 2014 to 2015 and from 2016 to 2017), which is consistent with the total ISA results estimated from the ISA% distribution maps generated in this study (R^2^ = 0.9).

Previous studies have reported that pixels are often mixed by impervious surfaces and other land covers, even at a spatial resolution of 30 m [17,64,65,66]. Therefore, underestimation or overestimation of the total ISA can occur when using the GAIA dataset. In contrast, the ISA% maps of this study were generated at a coarse spatial resolution of 1 km, whereas the mixture analysis technique allowed us to estimate ISA% on a sub-pixel scale, thus yielding a more accurate ISA% estimation (see Figure 6).

### 4.3. Watershed Health Status in Indonesia

Even in 2021, 88% (674 out of 766) of Indonesia’s watersheds are still in the no impact category, and only 0.9% are in the impacted (6 out of 766) and degraded (1 out of 766) categories (Figure 8a). However, the total ISA in Indonesia has almost tripled from 3687.4 km^2^ in 2003 to 10,505.5 km^2^ in 2021, and 85% of the latter is in the stressed category (Table 3 and Figure 8b). Additionally, 72% of Indonesian watersheds did not have ISA in 2003, but this value has decreased to 42% in 2021 (Figure 8c). All the above results indicate that land development in Indonesia was mainly in rural areas rather than urban areas, and categorical changes will occur in the future.

The annual ISA% distribution maps generated in this study provide us an opportunity to adequately assess the health status of Indonesian watersheds from year to year using Schueler’s criteria. We hope that a classification map of the watershed health status will help policymakers and improve management of these watersheds in the future.

## 5. Conclusions

In this study, we developed a method to estimate ISA% by combining daytime (MODIS-NDVI) and nighttime (radiance-calibrated DMSP-OLS-NTL and SNPP-VIIRS-DNB-NTL) satellite data. The developed method performed well from low ISA% (rural) to high ISA% (urban) values, with an RMSD value of 0.52 km^2^, a MAPD value of 16.20%, and a Bias of −0.08 km^2^. In addition, since the developed method uses only satellite data as input, it can be easily implemented in other regions. However, the relationship between ISA% and NTL needs to be rebuilt due to differences in light use efficiency and economic development in different regions. We also found that the health status of Indonesian watersheds is currently not critical, as 88% of Indonesian watersheds are still in the no impact category in 2021. Nevertheless, the total ISA in Indonesia increased significantly from 2003 to 2021 (i.e., from 3687.4 km^2^ to 10,505.5 km^2^), and most of the increased ISA was in the low impact level categories (i.e., stressed and no impact). Therefore, without proper management of these watersheds, negative categorical changes in Indonesian watersheds could be considered in the future.

## Figures and Tables

**Table 1:** Datasets used in this study.

Data	Year	Data Model	Spatial Resolution	Source
MODIS NDVI (MOD13A2)	2003, 2004, 2006, 2010, 2012–2021	Raster	1 km	LPDAAC/NASA ^a^
DMSP-OLS annual composites	2003, 2004, 2006, 2010	Raster	1 km	NOAA/NGDC ^b^
SNPP-VIIRS-DNB annual composites	2012–2021	Raster	750 m	NOAA/NGDC ^c^
Watershed polygon	2013	Vector	-	HydroSHEDS v1 ^d^
Google Earth image	2003, 2004, 2006, 2010, 2012–2018	Raster	0.5 m	Google Earth

^a^ https://ladsweb.modaps.eosdis.nasa.gov/archive/allData/6 accessed on 1 May 2019. ^b^ https://eogdata.mines.edu/products/dmsp/ accessed on 1 April 2021. ^c^ https://eogdata.mines.edu/products/vnl/ accessed on 1 April 2021. ^d^ www.hydrosheds.org accessed on 1 September 2020.

**Table 2:** Coefficients for relationships between ISA% and NTL_corrected_ for each year.

Year	a	b	c	d	R^2^	RMSD
2003	−0.0045	0.1563	2.7992	0.1341	0.996	1.91
2004	−0.0043	0.1479	2.9148	−0.3508	0.998	1.58
2006	−0.0014	0.0454	3.3163	−0.7795	0.998	1.58
2010	−0.0011	0.0343	3.1720	−0.4675	0.997	1.88
2012	−0.0034	0.1114	3.1167	−1.3497	0.997	1.82
2013	−0.0022	0.0836	2.6518	−0.2228	0.995	2.20
2014	−0.0033	0.1373	2.1018	0.1327	0.998	1.40
2015	−0.0015	0.0609	2.3866	−0.5878	0.998	1.58
2016	−0.0011	0.0493	2.1517	0.0846	0.996	2.05
2017	−0.0011	0.0426	2.5180	0.2164	0.998	1.44
2018	−0.0004	0.0031	2.9631	0.0917	0.996	1.93
2019	−0.0006	0.0263	2.3739	0.9115	0.995	2.25
2020	−0.0001	−0.0044	2.7183	0.4257	0.995	2.22
2021	−0.0001	−0.0180	3.3164	−0.1218	0.991	2.99

**Table 3:** Total ISA for each year in Indonesia obtained from the proposed method and the GAIA dataset, respectively.

Year	Estimated ISA (km^2^)	GAIA (km^2^)
2003	3687.35	4350.91
2004	3776.15	4465.25
2006	3912.47	4758.85
2010	4083.74	5270.11
2012	4061.37	5706.70
2013	4378.33	6089.81
2014	4671.75	6645.60
2015	4788.95	7537.54
2016	4931.67	8081.03
2017	6623.52	8234.18
2018	8466.58	8398.27
2019	9065.70	
2020	9397.09	
2021	10,505.50	

## Data Availability

Not applicable.

## Supplementary Materials

The following supporting information can be downloaded at: https://www.mdpi.com/article/10.3390/s23104975/s1, Figure S1: Statistics of NTLcorrected values for 10 non-vegetation fraction groups ranging from 1% to 100% divided into deciles from 2003 to 2021. For each group, the 5%, 25%, 50%, 75%, 95% percentiles are shown; Figure S2: Distribution maps of ISA% in Indonesia from 2003 to 2021; Figure S3. Distribution maps of watershed health status in Indonesia from 2003 to 2021.

## Abbreviation

CV	Coefficient of Variation
DMSP-OLS	Defense Meteorological Satellite Program-Operational Linescan System
DNB	Day and Night Band
EANTLI	Enhanced vegetation index Adjusted Nighttime Light Index
EOG	Earth Observation Group
EVI	Enhanced Vegetation Index
FCLS	Fully Constrained Least Squares
GAIA	Global Artificial Impervious Area
GDP	Gross Domestic Product
HydroSHEDS	Hydrological data and maps based on SHuttle Elevation Derivatives
ISA	Impervious Surface Area
ISA%	Impervious Surface Area Percentage
MAPD	Mean Absolute Percentage Difference
MNF	Minimum Noise Fraction
MODIS	Moderate Resolution Imaging Spectroradiometer
NDVI	Normalized Difference Vegetation Index
NOAA	National Oceanic and Atmospheric Administration
NTL	Nighttime Light
RMSD	Mean Square Difference
SMA	Spectral Mixture Analysis
SNPP	Suomi National Polar-orbiting Partnership
TMA	Temporal Mixture Analysis
TSOL	Total Sum of Light
VIIRS	Visible Infrared Imaging Radiometer Suite
