# Supplementary material for: Evaluation of the Health Status of Indonesian Watersheds Using Impervious Surface Area as an Indicator
Source: Sensors (Basel). 2023 May 22;23(10):4975. doi: 10.3390/s23104975 (PMC10220651; doi:10.3390/s23104975)
Supplement: Supplementary file 1 [file sensors-23-04975-s001.zip › sensors-2385394-supplementary.pdf]

Evaluation of the Health Status of Indonesian Watersheds Using Impervious Surface Area as an Indicator

Rossi Hamzah [1,2] and Bunkei Matsushita [3,*]

1 Graduate School of Life and Environmental Sciences, University of Tsukuba, Tennoudai 1-1-1, Tsuku-ba 305-8572, Japan; s1730209@u.tsukuba.ac.jp
2 Research Center for Remote Sensing, National Research and Innovation Agency (BRIN), Bogor, West Java 16911, Indonesia
3 Faculty of Life and Environmental Sciences, University of Tsukuba, Tennoudai 1-1-1, Tsukuba 305-8572, Japan

* Correspondence: matsushita.bunkei.gn@u.tsukuba.ac.jp; Tel.: +81-29-853-7190

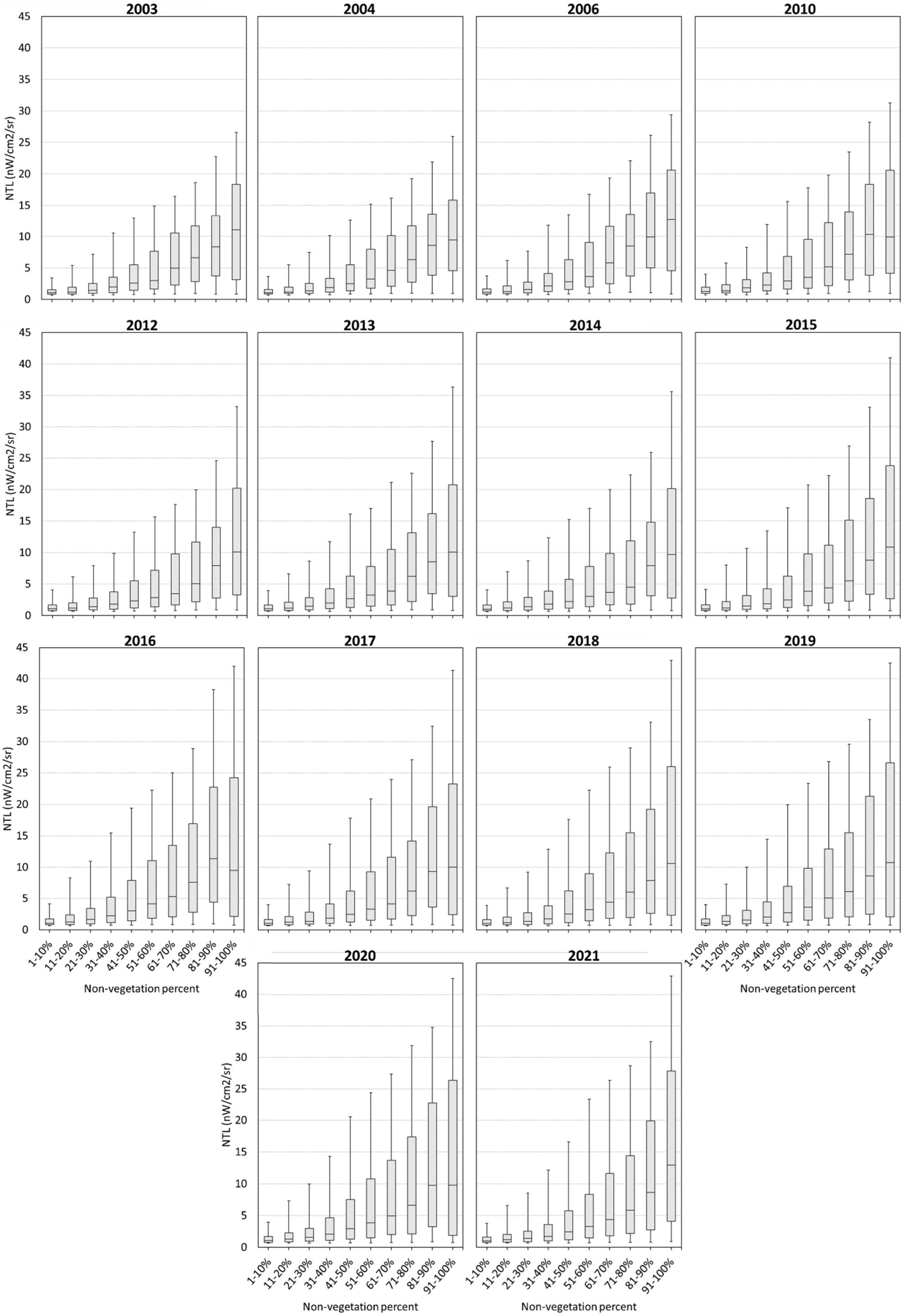

Figure S1. Statistics of $NTL_{corrected}$ values for 10 non-vegetation fraction groups ranging from 1% to 100% divided into deciles from 2003 to 2021. For each group, the 5%, 25%, 50%, 75%, 95% percentiles are shown.

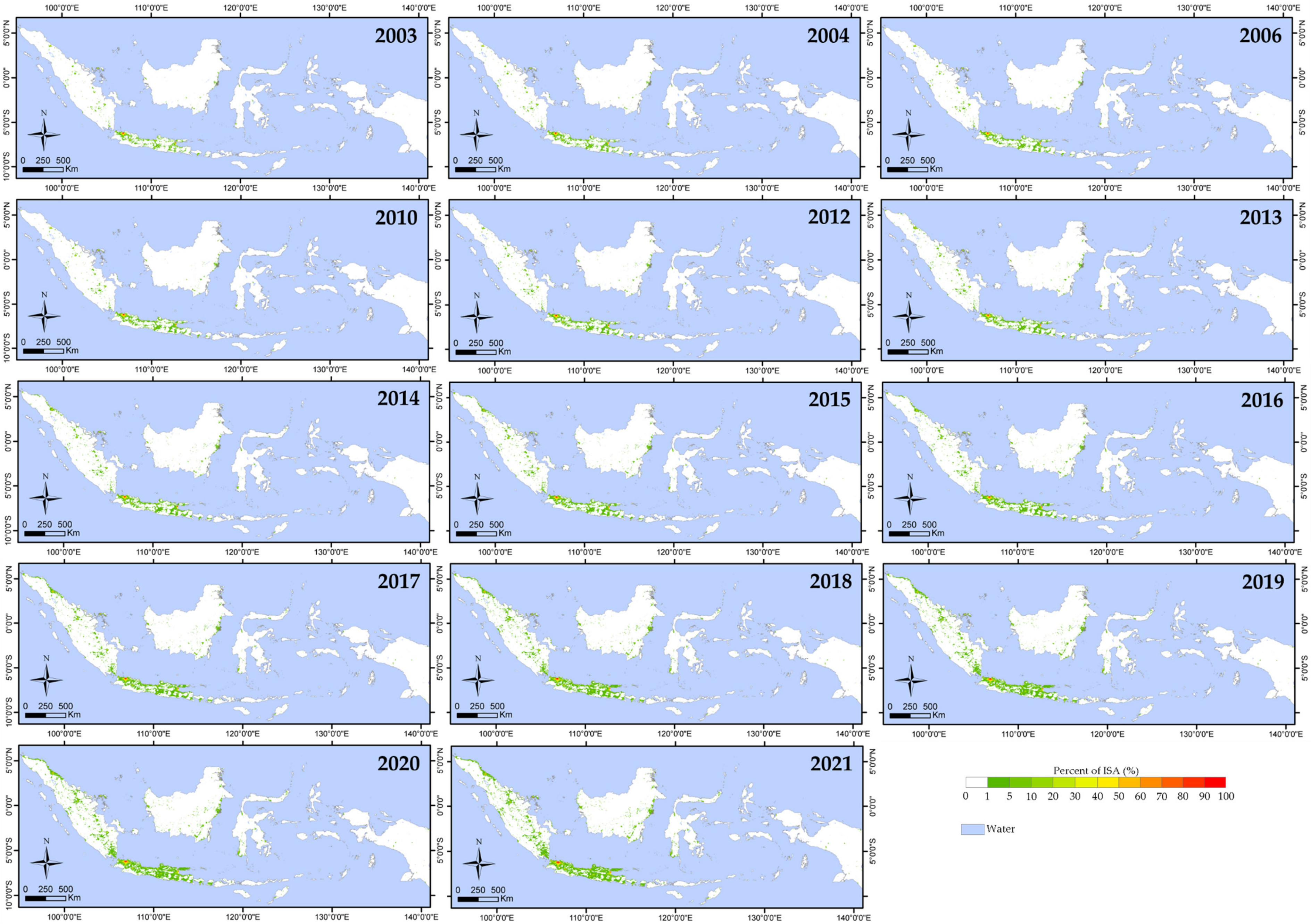

Figure S2. Distribution maps of ISA% in Indonesia from 2003 to 2021.

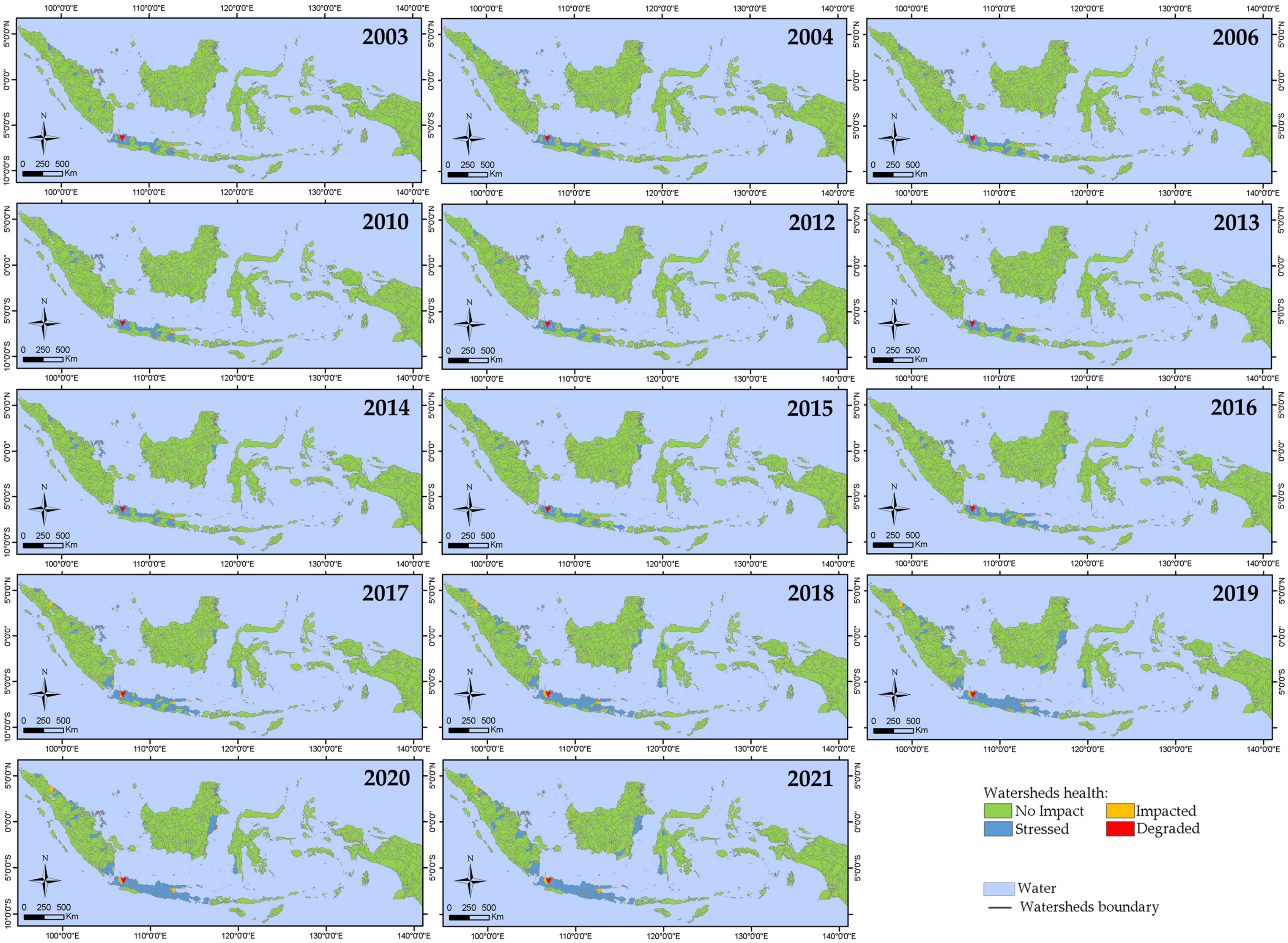

Figure S3. Distribution maps of watershed health status in Indonesia from 2003 to 2021.
